# Acute exposure to the conspecific alarm substance and its intergenerational consequences in *Astyanax bimaculatus* (Linnaeus, 1758)

**DOI:** 10.1007/s10695-026-01763-4

**Published:** 2026-08-05

**Authors:** Jeane Rodrigues, Eduardo Albuquerque, Evagno Ferreira, Paulo de Souza Jesus, Vanessa Coimbra, Emanuelly Aires, Bianca Lima de Sousa, Maria Clara Rosa-Silva, Otávio Lacerda, Caio Maximino, Diógenes Henrique de Siqueira-Silva

**Affiliations:** 1 Research Group of Studies On the Reproduction of Amazon Fish (GERPA/LANEC), Biology Faculty (FACBIO), University Federal of South and Southern of Pará (Unifesspa), Av. Dos Ipês, Marabá, Pará Zip-Code, S/N 68507-590 Brazil; 2 Postgraduate Program in Biodiversity and Biotechnology (BIONORTE)of the , Federal University of Pará (UFPA), Rua Augusto Corrêa, Belém, PA Brazil; 3 Postgraduation Program in Animal Reproduction in the Amazon (ReproAmazon)of the , Federal Rural University of the Amazon (Ufra), Federal University of Pará (UFPA), Av. Presidente Tancredo NevesTerra Firme Zip-Code: 66.077-830, Belém, PA 2501 Brazil; 4 University of South Bohemia in České Budějovice, Faculty of Fisheries and Protection of Waters, South Bohemian Research Center of Aquaculture and Biodiversity of Hydrocenoses, Research Institute of Fish Culture and Hydrobiology, Zátiší 728/II, Vodňany, 389 25 Czech Republic

**Keywords:** CAS, Embryology, Offspring, Tetra fish

## Abstract

The conspecific alarm substance (CAS), a chemical compound likely based on glycosaminoglycans, purines, and nitrogen oxide-based pterins, is a natural stressor released by specialized epidermal cells damaged by injury or attack in fish, inducing hormonal and behavioral changes in conspecifics. Its effects in the reproductive context are poorly understood, but previous studies have demonstrated earlier spawning in broodstock exposed to CAS and an influence on the larval length of the offspring, potentially linked to increased cortisol transfer. In this study, 53 sexually mature specimens of *Astyanax bimaculatus* (18 males and 35 females) were exposed to CAS for 6 min, before hormonal induction by intraperitoneal injection of carp pituitary extract solution. The CAS substance was extracted by injuring the skin of donor conspecific animals, by superficial cuts of the epidermis, using scissors. After hormonal induction, the animals were paired for courtship. Following semi-natural reproduction, we assessed spawning time, embryonic development, hatching rate, larval anomalies in the first 18 h, initial larval linear morphometry in the first 72 h, and offspring survival for 7 days. The exposed animals spawned 1 h earlier than the non-exposed breeders. Despite this, embryonic development, hatching rate, larval anomalies, and survival were similar for the offspring resulted from the exposed and non-exposed groups, and the offspring of stressed breeders showed significantly shorter total length, improved cephalic development, a thicker dorsal muscle region, and greater yolk consumption 48 h after hatching. The scarcity of research on the physiological consequences of CAS on fish reproduction limits the interpretation of intergenerational findings. The precocity of gamete release suggests CAS as a potential trigger of reproduction in the species, but the results in the progeny remain inconclusive. Further studies, including endocrine assessments, are needed to confirm or refute the intergenerational patterns induced by CAS.

## Introduction

The conspecific alarm substance (CAS) is composed of a set of odoriferous chemical substances that likely include glycosaminoglycans, such as chondroitin sulfate, as well as purines and nitrogen oxide–based pterins (Maximino et al. 2019). These substances are produced by specialized cells called club cells or claviform cells in the epidermis of fish species (Pfeiffer 1977; Saadatfar and Shahsavani 2007; Saadatfar, et al. 2010; Shooraki et al. 2018), such as those belonging to the superorder Ostariophysi (Pfeiffer 1977).

*Club* cells do not possess channels for the spontaneous release of CAS (Pfeiffer 1977). The release of this substance occurs when the animal’s skin is injured (Von Frisch 1941), which is associated with attacks or predation risk. Once released, CAS is perceived by sensory neurons present in the epithelial tissue of the olfactory channels of conspecifics (Von Frisch 1941; Maximino et al. 2019). As it is a natural stressor agent, it triggers an increase in cortisol levels shortly after exposure in fish species (Mathuru et al. 2012; Schirmer et al. 2013; Silva et al. 2015; Abreu et al. 2017), which in turn is translated into antipredator alarm behavioral responses (Rehnberg and Schreck 1987; Rehnberg et al. 1987; Toa et al. 2004).

Studies involving the presence of these cells, exposure, and alarm behavior in the genus *Astyanax* have already been conducted. There are reports of the presence of *club* cells in species such as *Astyanax fasciatus* (Fricke 1987), *Astyanax jordani* (Pfeiffer 1963), and *Astyanax bimaculatus* (Pfeiffer 1963; Rodrigues et al. 2023), as well as exposure alarm reactions (Pfeiffer 1963; Rodrigue et al. 2023). Our studies with the species *A. bimaculatus* began with the evaluation of the presence of these cells and alarm reaction to acute CAS exposure (Rodrigues et al. 2023).

Exposure to CAS in the reproductive context of fish species remains largely unexplored. It is known that both acute (Rodrigues et al. 2023) and prolonged (Pollock et al. 2005; Jung and Tonn 2011) exposure to CAS trigger early spawning, indicating possible physiological effects on breeders and, consequently, on their gametes. In the intergenerational context, studies are even scarcer. It is only known that maternal exposure causes effects on larval development in the species *Pimephales promelas* (Jung and Tonn 2011) and that paternal stress, induced by CAS exposure, can be transferred to the offspring and alter their behavior (Ord and Watt 2022).

The early life stages of a fish are determinant for its development and survival. They are particularly sensitive to physiological changes and internal or environmental stress. Embryos incubated in cortisol exhibit lower overall growth rates (Mccormick and Nechaev 2002). In the larval stage, molecules involved in stress responses, such as cortisol, expressed in cells of the hypothalamic-pituitary-interrenal (HPI) axis in adults, are present in several other organs of the body and likely play the same role, which can be particularly important for their survival (Pederzoli and Mola 2016). In carp (*Cyprinus carpio*), the HPI endocrine stress axis is functional already at larval hatching to adjust its physiology to the constantly changing environment (Flik et al. 2006); however, in European sea bass (*Dicentrarchus labrax*), the cortisol stress response becomes complete only at 45 days of life (Tsalafouta et al. 2014). Thus, in the early life stages of fish, other organs and tissues may substitute for or cooperate with the HPI axis in the stress response (Pederzoli and Mola 2016).

The present study aimed to evaluate the intergenerational effects on *A. bimaculatus* embryos and larvae originating from broodstock acutely exposed to a conspecific alarm substance. *Astyanax bimaculatus* is considered one of the most important species in the genus *Astyanax* (Vilela and Hayashi 2001), being widely prevalent in South America, in the Panama and Amazon basins (Lima et al. 2003). This species is commonly used in sport fishing (Barbieri et al. 1982) and is appreciated as a snack (Rezende et al. 2005). It is also used as an ornamental fish due to its peaceful behavior and easy reproduction in captivity (Rezende et al. 2005). Such attributes make it an important ally for sustainable food production and the socioeconomic development of rural communities (Fonseca et al. 2017). Additionally, it plays a role in the natural biological control of organisms such as the phlebotomine *Simulium pertinax* (Murayama 2010) and serves as a food source for species like the saicanga (*Oligosarcus longirostris*) and the traíra (*Hoplias malabaricus*) (de Fatima et al. 2001).

## Material and methods

### Ethical considerations

The project was registered in the National System for the Management of Genetic Heritage and Associated Traditional Knowledge of the Ministry of the Environment, Brazil (registration n° A0955F0), and the use of the species followed the standards issued by the National Council for the Control of Animal Experimentation (CONCEA), with approval from the Animal Use Ethics Commission (CEUA) of the Federal University of South and Southeast Pará (Unifesspa) (Record N° 23,479.010692/2021–16).

#### Obtaining and acclimation of animals

Specimens of *A. bimaculatus* were obtained from a local producer in Marabá, Pará (5°21′04.7″ S 49°05′14.5″ W). The animals were placed in 40-L plastic bags equipped with portable aerators (VIGOAR) and transported to the Neuroscience and Behavior Laboratory (LaNeC) at the Federal University of South and Southeast Pará (Unifesspa). In the laboratory, the animals were acclimated for a minimum period of 15 days in two 100-L water tanks: one for the control group and another for the CAS-exposed group. Both tanks had constant mechanical aeration, biological filtration, artificial vegetation (de Souza et al. 2024), and a 12-h light/12-h dark photoperiod. Feeding was provided twice daily with commercial feed GUABI, Pirá line (28% crude protein, 5% fat, 300 mg/kg of vitamin C, and 87.5 IU/kg of vitamin E) and captive-bred *Tenebrio molitor* larvae. Water physical and chemical parameters were measured weekly in both tanks. The control tank averaged 0.8 ± 0.4 ppm ammonia, 6.5 ± 0.1 ppm dissolved oxygen, 1.1 ± 0.9 ppm nitrite, temperature of 26.7 ± 0.8 °C, pH 7 ± 0, conductivity (EC) 122 ± 8.5, and hardness of 60.0 ± 5.7 ppm. The tank with animals raised for CAS exposure averaged 0.3 ± 0 ppm ammonia, 6.3 ± 0.1 ppm dissolved oxygen, 0.6 ± 0.5 ppm nitrite, temperature of 26.6 ± 0.1 °C, pH 7 ± 0, conductivity (EC) 120.5 ± 12.0, and hardness of 61.0 ± 4.2 ppm. Hardness, ammonia, and nitrite analyses were performed using commercial colorimetric kits (LabconTest®), following the manufacturer’s instructions. Parameters such as pH, temperature, and conductivity were measured with the Hanna HI-98194 equipment, and dissolved oxygen with the Generic equipment, all previously calibrated.

#### Extraction and preparation of conspecific alarm substance

The preparation of the CAS-containing solution followed the procedures of Rodrigues et al. (2023) for the species *A. bimaculatus*. The protocol consisted of euthanizing males and females with a lethal dose of eugenol solution (20 mL of eugenol in 180 mL of 100% alcohol, BIODINÂMICA). Following this, the scales of the euthanized animals were removed for dissection and total epidermal collection. A 0.8 g portion of skin was removed (avoiding blood contamination), chopped with scissors (150 cuts), and immersed in 8 mL of distilled water (0.1 g:1 mL) per exposed animal. The solution was agitated every 5 min, followed by 5 min of rest, for 30 min. Subsequently, the solution was filtered, and 7 mL of CAS solution was collected using a micropipette and stored in graduated tubes (*Falcon*, 50 mL) under refrigeration (4 °C).

#### Exposure to conspecific alarm substance (CAS)

Acute exposure to CAS was performed based on our previous study with this species (Rodrigues et al. 2023). Eighteen sexually mature males and 36 sexually mature females were selected and divided into two large groups, with three replicates each. In the treatment group, 9 males (body weight 3.1 ± 0.6 g, total length 7 ± 0.3 cm, and standard length 5.5 ± 0.4 cm) and 18 females (body weight 3.2 ± 1 g, total length 6.3 ± 0.7 cm, and standard length 5.3 ± 0.6 cm) were placed in aquariums with 1 L of water per gram of body weight, based on Lima-Maximino et al. (2020) experiments using zebrafish. These animals were exposed to 18.9 mL of CAS solution for 6 min. In the control group, 9 males (body weight of 3.8 ± 1.6 g, total length of 7 ± 0.7 cm, and standard length of 5.5 ± 0.6 cm) and 18 females (body weight of 3.8 ± 2 g, total length of 6.5 ± 1.1 cm, and standard length of 5.5 ± 0.9 cm), kept in aquariums with the same water volume ratio, were exposed for 6 min to 18.9 mL of distilled water, used as a vehicle in the previous treatment. Before and after animal exposure to the alarm substance and distilled water (CAS preparation vehicle), water physical and chemical parameters (hardness, pH, ammonia, temperature, and dissolved oxygen) were measured and were in accordance with reference values for the tests, indicating ideal aquatic conditions for freshwater fish during experimentation (Table 1).

**Table 1:** Average values of physicochemical water parameters from the experimental unit of *A. bimaculatus* broodstock exposed and no-exposed to conspecific alarm substance

Parameters	Reference value	Pre-exposure		Post-exposure	
		Control	CAS	Control	CAS
Ammonia (ppm)	0–0.04	0.01 ± 0	0.03 ± 0	0.01 ± 0	0.02 ± 0
O2 (ppm)	4–11	6.3 ± 0.1	6.5 ± 0.1	8.7 ± 2	8.4 ± 2.2
Nitrite (ppm)	0–0.25	0.0 ± 0	0.0 ± 0	0.0 ± 0	0.0 ± 0
Temperature (°C)	24–26	26.5 ± 0.2	26.6 ± 0.6	24.6 ± 0.3	24.9 ± 0.6
pH	5.5–8.5	7.1 ± 0.2	7.1 ± 0.2	7.2 ± 0.2	7.2 ± 0.1
Hardness (ppm)	60–150	60.0 ± 5.7	61.0 ± 4.2	52.0 ± 1.4	64.0 ± 2.8
Conductivity (µS/cm)	50–500	120.5 ± 12	122.0 ± 8.5	103.5 ± 2.1	127.5 ± 4.9

#### Hormonal induction and semi-natural fertilization

Six minutes after exposure, animals from each group were transferred to new 9-L aquaria with CAS-free water. They were then subjected to in vitro reproduction. For this, they were anesthetized in 1 mL eugenol solution (20 mL eugenol in 180 mL 100% ethanol, BIODINÂMICA) diluted in 800 mL water and then submitted to hormonal induction with crude carp pituitary extract (CPE). The injection was performed intraperitoneally below the pectoral fin of the specimens, in the cephalocaudal direction, using an insulin syringe (0.5 mL). Females received two doses of 0.1 mL CPE, with a 12-h interval between them, to promote oocyte maturation and release, respectively. Spermiation in males was induced with a single dose of 0.05 mL CPE, at the time of the second dose application in females (Rodrigues et al. 2023).

After hormonal induction, the broodstock were kept in their aquaria with constant aeration for a period of 6 h for reproductive courtship, gamete release, and semi-natural fertilization. A submerged net separated them from the bottom of the aquarium, where the embryos were deposited. This protocol is used to prevent the ingestion of embryos by the broodstock themselves, given that this is a species without parental care.

#### Progeny test

After fertilization, the spawning time, embryonic development, hatching rate, rate of morphological abnormalities, and linear morphometry of the larvae from each group were evaluated in triplicate. Spawning time was calculated from the first hormonal injection, in hours post-induction. Embryonic development evaluation was adapted from Miranda et al. (2023) for the species *A. bimaculatus*. The process consisted of collecting embryos every 10 min post-fertilization (pf) until reaching 2 h of development; every 20 min pf until reaching 4 h of development; one collection after 1 h, reaching 5 h pf; and finally, collections every 2 h until larval hatching. The adaptation was based on a pilot experiment for this type of study and species. Collected embryos were fixed in 2.5% glutaraldehyde solution (0.2 M dibasic phosphate buffer, pH 7.4, ÊXODO CIENTÍFICA) for subsequent evaluation of embryonic stages.

The hatching rate was quantified from the selection of 200 viable embryos from each group. Larval morphological abnormality rate was assessed 18 h post-hatching (hph), with random collection of 17 larvae from each treatment and fixation in 2.5% glutaraldehyde solution (0.2 M dibasic phosphate buffer, pH 7.4). The analyses followed Pinheiro et al. (2021) protocol for another *Astyanax* species. Analyzed anomalies included head malformation, scoliosis, modified notochord, pericardial edema, deformed yolk sac, lack of pigmentation, short tail, and retarded growth, which were evaluated as present (1) or absent (0) and the frequency (%) of each anomaly in each group (control and CAS) using simple rule of three.

#### Initial linear larval morphometry

The morphometric analysis consisted of comparing 51 individuals per treatment, collected equitably (17 individuals) at 18, 48, and 72 h post-hatching (hph). During this period, the animals do not perform exogenous feeding, with their growth resulting from the consumption of yolk energy reserves. The specimens were fixed in 2.5% glutaraldehyde solution in 0.2 M dibasic phosphate buffer (pH 7.4).

Morphometric measurements were based on Coimbra et al. (2025), with adaptations for this species and study regarding dorsal muscle height (DMH). Briefly, fixed larvae were photographed under a stereomicroscope (LUMEN) with universal adapter (OPTICA) and digital camera for linear morphometric measurement in *ImageJ software* (version bundled with 64-bit Java 8).

**Fig. 1:**
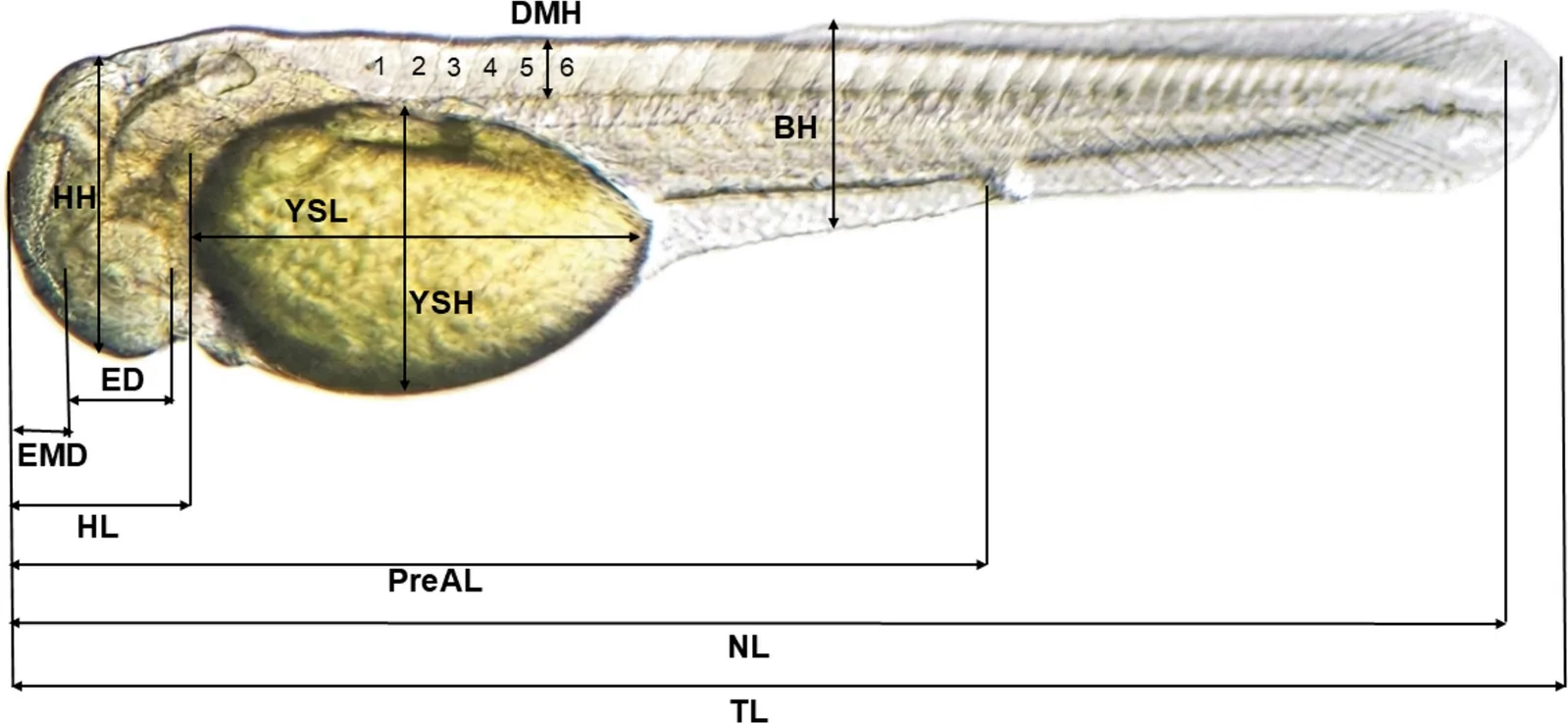
Schematic diagram of morphometric measurements of *A. bimaculatus* larvae from broodstock exposed to CAS. Total length (TL), eye-to-mouth distance (EMD), eye diameter (ED), head length (HL), head height (HH), pre-anal length (PreAL), notochord length (NL), body height (BH), dorsal muscle height (DMH), yolk sac length (YSL), and yolk sac height (YSH). Numbers 1, 2, 3, 4, 5, and 6 (myomeres). Original image, enhanced with artificial intelligence (GEMINI)

The body measurements analyzed, expressed in millimeters, were total length (TL), eye-to-mouth distance (EMD), eye diameter (ED), head length (HL), head height (HH), pre-anal length (PreAL), notochord length (NL), body height (BH), dorsal muscle height (DMH), measured between the fifth and sixth myomere, yolk sac length (YSL), and yolk sac height (YSH) (Fig. 1).

#### Initial larval survival

After hatching, 100 larvae from CAS-exposed broodstock and 100 larvae from control group broodstock, along with their respective replicates, were placed in six 3-L aquaria for 7 days, with water quality parameters monitored (Table 2). For the first 3 days, larvae had their aquarium water completely renewed daily, and quality parameters such as temperature, pH, hardness, conductivity, and dissolved oxygen (O2) were measured. During this period, the surviving larvae were counted to assess the initial and endogenous effects of parental exposure. Dissolved nitrite and ammonia were not measured due to total water renewal.

**Table 2:** Average values of physical and chemical water quality parameters during the culture of larvae from *A. bimaculatus* broodstock exposed and no-exposed to conspecific alarm substance. *NA*, not assessed

Group	Day	*T* (°C)	pH	Hardness	Conductivity	O2	Nitrite	Ammonia
		(24–26)	(5.5–8.5)	(60–150)	(50–500)	(4–11)	(0–0.25)	(0–0.04)
**Control**	0	22.9 ± 0.4	7.5 ± 0	52 ± 0	103 ± 0	7.5 ± 0	0.0 ± 0	0.0 ± 0
	1	24.8 ± 0.4	6.9 ± 0.1	51 ± 1	103 ± 0.6	9.2 ± 0.3	NA	NA
	2	26.5 ± 0.7	7.2 ± 0.1	51 ± 1	102 ± 0.6	9.7 ± 0	NA	NA
	3	25.9 ± 0.7	7.02 ± 0	51 ± 1	101 ± 0	9.5 ± 0.4	NA	NA
	4	24.9 ± 0.6	6.9 ± 0	52 ± 0	104 ± 0.6	9.5 ± 0.1	0.0 ± 0	0.0 ± 0
	5	25.9 ± 0.3	6.5 ± 0.3	NA	NA	NA	0.25 ± 0	0.02 ± 0
	6	25.4 ± 0.6	6.3 ± 0.4	NA	NA	NA	0 ± 0	0 ± 0
	7	25.8 ± 0.1	6.6 ± 0	NA	NA	NA	0.25 ± 0	0.01 ± 0
**CAS**	0	22.6 ± 0.2	7.5 ± 0	52 ± 0	103 ± 0	7.5 ± 0	0.0 ± 0	0.0 ± 0
	1	24.5 ± 0.2	6.9 ± 0.1	53 ± 2	105 ± 3.2	9 ± 0.4	NA	NA
	2	26.3 ± 0.3	6.9 ± 0.2	52 ± 3	101 ± 1	10.1 ± 0	NA	NA
	3	25.7 ± 0.3	7.1 ± 0	51 ± 1	102 ± 0.6	9.3 ± 0.1	NA	NA
	4	24.5 ± 0.3	7 ± 0.02	53 ± 0	106 ± 0.6	9.3 ± 0.1	0.0 ± 0	0.0 ± 0
	5	25.2 ± 0	6.5 ± 0.1	NA	NA	NA	0.33 ± 0	0.02 ± 0
	6	25 ± 0	6.3 ± 0.1	NA	NA	NA	0.08 ± 0	0.03 ± 0
	7	25.8 ± 0.1	6.5 ± 0	NA	NA	NA	0.25 ± 0.3	0.01 ± 0

At the end of the third day, due to observation of open mouth and residual yolk sac, exogenous feeding was implemented with 8.75 mg commercial feed (50% crude protein, Aqua® line), based on amounts used for this species in another laboratory study (de Sousa et al. 2024). From the fourth day onward, partial water exchange was performed every 24 h and total exchange every 48 h. On the seventh day, larvae were counted again to evaluate late effects of parental exposure on offspring survival.

#### Statistical analyses

Statistical analyses were performed using R software (version 4.4.2), adopting a significance level of 5% (*p* < 0.05). Spawning time and larval hatching time were evaluated using the nonparametric Mann–Whitney test (Wilcoxon rank-sum) for group comparisons.

Hatching rate and larval morphological anomalies were analyzed using a generalized linear model (GLM) with binomial distribution and logit link function. For larval morphological anomalies, recorded as binary data (0 = absent; 1 = present), frequencies and percentages of each anomaly were previously calculated for both groups (control and CAS). Group comparisons were performed using Fisher’s exact test, considering the categorical nature of the data.

Linear larval morphometry was evaluated using two-way ANOVA, considering parental treatment (control and CAS-exposed) and time post-hatching (18, 48, and 72 h) as fixed factors. Prior to analysis, assumptions of residual normality (Shapiro–Wilk test) and homogeneity of variances ((Levene’s test) were verified. In case of significant results, Tukey’s HSD post-hoc test was applied for multiple comparisons. For variables violating assumptions, the nonparametric Kruskal–Wallis test was applied, followed by Dunn’s post-hoc test with Bonferroni correction when necessary.

Larval survival was analyzed using the Kaplan–Meier method, with data collected on days 0, 3, and 7 post-hatching. Survival curves between groups were compared using the log-rank test (Mantel-Cox), considering a significance level of *α* = 0.05.

All results are presented as mean ± standard deviation, except for the larval abnormality rate, which is also presented as a percentage (%).

## Results

### Spawning and embryonic development

Gamete release occurred semi-naturally in both groups, without the need for extrusion. The CAS group spawned on average 1 h earlier (7:51 ± 4 h) than the control group, which spawned at 8 h 56 min ± 3 h after the first hormonal induction, but the groups were statistically similar (*W* = 3, *p* = 0.6625). Embryos from CAS-exposed broodstock developed similarly to the control group but showed subtle differences in somite number during late segmentation and chorion rupture, culminating in hatching on average 54 min later than the control group, which hatched at 17:06 ± 35 min post-fertilization (Table 3, *W* = 6.5; *p* = 0.49). It is worth noting, however, that this difference was not statistically significant, despite the observed biological shift.

**Table 3:** Embryonic development of *Astyanax bimaculatus* exposed and no-exposed to CAS

Time (h)	Phase	Stage		Figure
		Control	CAS	
00:00	Zygote	Zygote	Zygote	a
00:10	Cleavage	1 cell	1 cell	b
00:20		2 cells	1 cell	b–c
00:30		4 cells	2 cells	c–d
00:40		8 cells	4 cells	d–e
00:50		16 cells	8 cells	e–f
01:10		16 cells	16 cells	f
01:30		32 cells	32 cells	g
01:40		64 cells	64 cells	h
01:50	Blastula	128 cells	128 cells	i
02:00		128 cells	256 cells	i–j
02:20		256 cells	512 cells	j–k
03:00		512 cells	Dome	k–l
03:20		Dome	Dome	L
03:40	Gastrula	50% epiboly	50% epiboly	M
04:00		75% epiboly	75% epiboly	n
05:00		Blastopore	95% epiboly	o
07:00	Neurula	100% epiboly	100% epiboly	p
09:00	Segmentation	8 somites	10 somites	q
11:00		13 somites	16 somites	r
13:00		19 somites	20 somites	r–s
15:00		26 somites	29 somites	r–s
17:00		Hatching	33 somites	s–t
18:00			Hatching	t

### Zygote stage

The zygote stage, defined by the differentiation between the animal pole (blastodisc) and vegetal pole, lasted up to 10 min post-fertilization (pf) for both groups (control and CAS).

### Cleavage stage

The cleavage stage, characterized by meroblastic discoidal divisions into 2, 4, 8, 16, 32, and 64 blastomeres consecutively, lasted 1 h 40 min post-fertilization (pf) for both groups.

### Blastula stage

This phase began with cell duplication from the sixth cleavage, initiating the sequence of 128, 256, and 512 blastomeres, and ended with the dome stage, characterized by thousands of blastomeres forming layers on top of the vegetal pole, which occurred at 3 h 20 min post-fertilization (pf) for embryos from control broodstock and 3 h pf for embryos from CAS-exposed broodstock.

### Gastrula stage

This phase is characterized by the onset of epiboly, where blastomeres gradually move toward the vegetal pole region. At 3 h 40 min post-fertilization (pf), a germinal ring is observable in both groups. The gastrula development phase lasted 1 h 20 min.

### Neurula stage

This phase is characterized by the complete envelopment of the embryo by the blastoderm, completing 100% epiboly. This phase occurred at 7 h post-fertilization (pf) for both groups.

### Segmentation stage

The segmentation stage, characterized by the appearance of somites, yolk, and internal and external organs of the embryo, extended until hatching. This phase began 9 h after fertilization, with the appearance of the first 8 ± 1 somites in the control group and 10 ± 0 somites in the CAS group embryos. It progressed to 12 ± 2 and 16 ± 2 somites at 11 h after fertilization and 19 ± 1 and 20 ± 1 somites at 13 h after fertilization in the control and CAS groups, respectively. Its completion occurred around 15 h after fertilization, with 26 ± 4 somites in the control group and, at 17 h after fertilization, with 33 ± 4 somites in the CAS group.

### Hatching stage

**Fig. 2:**
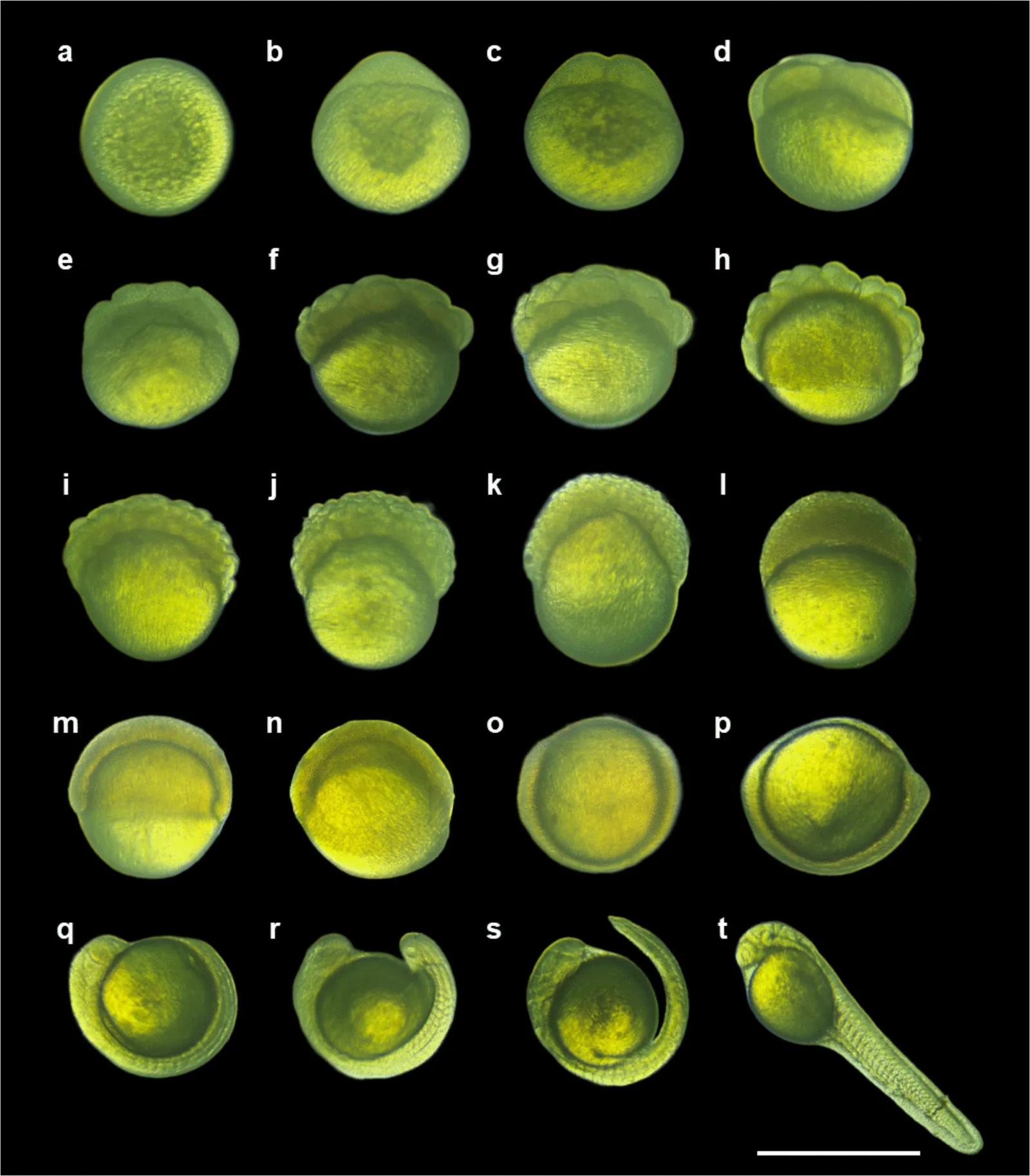
Embryonic development stages of *Astyanax bimaculatus* from broodstock exposed to CAS. **a** Zygote; **b** 1 cell; **c** 2 cells; **d** 4 cells; **e** 8 cells; **f** 16 cells; **g** 32 cells; **h** 64 cells; **i** 128 cells; **j** 256 cells; **k** 512 cells; **l** dome; **m** 50% epiboly; **n** 75% epiboly; **o** 95% epiboly; **p** 100% epiboly; **q** 8–10 somites; **r** 13–16 somites; **s** embryo with 19–33 somites and free tail; **t** newly hatched larva. Scale bar: 1 mm

Embryos showed free tails at 13 h pf in the control group and 15 h pf in the CAS group. Hatching of control group larvae occurred on average at 17 h 06 min ± 35 min pf (equivalent to 408 degree-hours), while in the CAS group, it occurred at 18 h ± 1 h 7 min pf (equivalent to 426 degree-hours). The evolution of embryonic development stages from *A. bimaculatus* broodstock over time, regardless of treatment, at an average temperature of 24 °C ± 1 °C is placed on Fig. 2.

### Hatching rate and larval abnormalities

The larval hatching rate showed no significant difference (*p* = 0.3589) between the control group with 46 ± 39 hatched larvae (23% of total) and the CAS group with 20 ± 2 hatched larvae (10% of total). Larvae from both groups exhibited normal morphology at 18 h post-hatching (hph).

### Larval linear morphometry

Bifactorial variance analysis revealed a significant effect of time post-hatching on the evaluated morphometric variables (*p* < 0.001), reflecting the expected ontogenetic growth of *A. bimaculatus* larvae during early development.

Significant parental treatment effects were observed for eye-to-mouth distance (EMD) (*p* = 0.0076), notochord length (NL) (*p* = 0.0001), and dorsal muscle height (DMH) (*p* = 0.0288). Additionally, significant interactions between parental treatment and post-hatching time were detected for total length (TL) (*p* = 0.0007), eye-to-mouth distance (EMD) (*p* = 0.0032), head length (HL) (*p* = 0.0042), head height (HH) (*p* = 0.0172), notochord length (NL) (*p* < 0.0001), and yolk sac height (YSH) (*p* = 0.0146), indicating that treatment effects varied over time.

Post-hoc comparisons showed no statistical differences between control and CAS groups at 18 hph. At 48 hph, greater yolk sac length (YSL) (*p* = 0.0125) was observed in the control group and greater dorsal muscle height (DMH) in the CAS group (*p* = 0.0465). At 72 hph, the CAS group exhibited significantly lower values than the control for total length (LT), notochord length (NL), and yolk sac height (YSH) and higher values for eye-to-mouth distance (EMD), head length (HL), and head height (HH) (*p* < 0.05) (Table 4).

**Table 4:** Mean and standard deviation of morphometric measurements (mm) of structures in *A. bimaculatus* larvae from broodstock exposed and no-exposed to CAS

	18 hpe		48 hpe		72 hpe	
	Control	CAS	Control	CAS	Control	CAS
**TL**	3.36 ± 0.14	3.35 ± 0.10	4.51 ± 0.37	4.60 ± 0.12	5.11 ± 0.27 a	4.71 ± 0.43 b
**BH**	0.45 ± 0.05	0.45 ± 0.06	0.67 ± 0.11	0.69 ± 0.07	0.75 ± 0.07	0.68 ± 0.11
**DMH**	0.11 ± 0.01	0.12 ± 0.01	0.13 ± 0.01 a	0.14 ± 0.01 b	0.14 ± 0.01	0.14 ± 0.01
**EMD**	0.08 ± 0.02	0.07 ± 0.02	0.11 ± 0.02	0.13 ± 0.03	0.17 ± 0.03 a	0.22 ± 0.06 b
**ED**	0.23 ± 0.02	0.24 ± 0.06	0.36 ± 0.03	0.36 ± 0.02	0.40 ± 0.03	0.38 ± 0.04
**HL**	0.35 ± 0.03	0.36 ± 0.03	0.94 ± 0.04	0.97 ± 0.04	1.15 ± 0.08 a	1.07 ± 0.12 b
**HH**	0.63 ± 0.03	0.66 ± 0.04	0.75 ± 0.06	0.75 ± 0.03	0.84 ± 0.06 a	0.79 ± 0.10 b
**PreAL**	2.10 ± 0.09	2.11 ± 0.06	2.39 ± 0.14	2.42 ± 0.10	2.70 ± 0.18	2.60 ± 0.31
**NL**	3.20 ± 0.14	3.20 ± 0.10	4.14 ± 0.35	4.21 ± 0.12	4.72 ± 0.29 a	4.08 ± 0.32 b
**YSL**	0.97 ± 0.05	0.95 ± 0.04	0.34 ± 0.11 a	0.28 ± 0.06 b	0.28 ± 0.05	0.28 ± 0.05
**YSH**	0.61 ± 0.04	0.63 ± 0.05	0.20 ± 0.08	0.16 ± 0.05	0.18 ± 0.04 a	0.23 ± 0.08 b

Different letters (a; b) in the same line indicate significant differences (ANOVA *p* < 0.05) between treatments

*TL* total length *BH* body height *DMH* dorsal muscle height *EMD* eye-to-mouth distance *ED *eye diameter *HL *head length *HH* head height *PreAL* pre-anal length *NL* notochord length *YSL* yolk sac length *YSH* yolk sac height

### Survival analysis

Kaplan–Meier survival analysis showed no statistically significant difference between control and CAS groups at 0, 3, and 7 days post-hatching, per log-rank test (*χ*^2^ = 3.7; df = 1; *p* = 0.054). At day 1 (0 h), survival was 100 ± 0 larvae in both groups. By day 3, control had 98 ± 3 live larvae vs. 87 ± 8 in CAS. By day 7, control had 31 ± 17 live larvae and CAS had 30 ± 6 live larvae.

## Discussion

Investigations into alarm substance presence in reproductive contexts are scarce, with little known about intergenerational CAS exposure effects in fish species. CAS is believed to induce stress in fish shortly after exposure, elevating cortisol levels (Stratholt et al. 1997; Contreras-Sanchez et al. 1998; McCormick 1998), which may act as a molecular regulator of spermatogenesis, such in zebrafish (*Danio rerio*) (Tovo-Neto et al. 2020). Cortisol can be transferred and gradually deposited in oocytes during vitellogenesis and, consequently, in embryos (Faught et al. 2016; Nesan and Vijayan 2016). Paternal stress from CAS exposure can be passed to juveniles, altering their behavior (Ord and Watt 2022), while maternal exposure affects oocyte release and viability (Rodrigues et al. 2023) and the development of fish larvae (Jung and Tonn 2011).

While the delayed hatching timing was not statistically significant, such variations warrant caution when interpreted ecologically. In the wild, fish spawning and hatching are often highly synchronized with environmental cues and prey availability. A baseline delay, even if subtle, could disrupt this synchrony and pose a disadvantage for larvae during the transition to exogenous feeding. Early spawning has already been observed in *Archocentrus nigrofasciatus* (Pollock et al. 2005) and *Pimephales promelas* (Jung and Tonn 2011) under prolonged CAS exposure. In acute CAS exposure, spawning of *A. bimaculatus* was also advanced by 20 min (Rodrigues et al. 2023). This physiological response may represent a pattern for fish species exposed to conspecific alarm substance.

Embryonic development and hatching times in this study aligned with expectations for *A. bimaculatus* under laboratory conditions at 24–25 °C, ranging from 16 to 17 h post-fertilization, consistent with prior reports (Weber et al. 2013). This lack of definitive embryonic disruption mirrors studies by Zhang et al. (2025), where direct CAS exposure in zebrafish embryos yielded no statistical developmental differences from controls. The subtle differences we observed in somite numbers and chorion rupture timing were not statistically significant and cannot be interpreted as definitive treatment effects. Further studies with a larger sample size are required, because existing literature demonstrates that stressed broodstock can exhibit elevated maternal-to-fetal cortisol transfer (Stratholt et al. 1997; Contreras-Sanchez et al. 1998; McCormick 1998; Faught et al. 2016; Nesan and Vijayan 2016). Interestingly, cortisol supplementation in teleost embryos has been shown to enhance the pulsation rates of somite cells during segment formation, as well as accelerate myotome contraction rhythms during muscle development and cardiac function (Mccormick and Nechaev 2002; Zhang et al. 2025).

The statistical similarity and arithmetic mean equivalence in hatching rates and initial larval survival between both groups in this study align with and complement findings of comparable survival in offspring from CAS-exposed and unexposed broodstock of *Archocentrus nigrofasciatus*, both during embryonic phases and the first 8 days of life (Pollock et al. 2005). No CAS-related morphological anomalies were found in directly exposed larvae or in larvae from exposed breeders in database searches or in this study. Stabell et al. (2010) also did not observe morphological alterations in CAS-exposed larvae, which is consistent with our findings.

First morphometric differences emerged at 48 hph, linked to energy consumption and muscle robustness. CAS larvae showed reduced yolk length indicating greater absorption, matching maternal stress-induced yolk depletion (McCormick 1998) and direct larval stressor exposure (Yabu et al. 2003; Werner et al. 2007; Shi et al. 2008; Lara and Vasconcelos 2021). Elevated dorsal muscle height (DMH) suggests cortisol-induced hypertrophy for the protection of internal organs, as observed in zebrafish larvae from broodstock fed cortisol, exhibiting larger muscle bundles (Rodrigues et al. 2025).

Additional morphometric differences emerged at 72 hph in CAS larvae, showing greater head length, head height, and eye-mouth distance, likely due to accelerated yolk consumption in the first 48 hph and compromised energy supply, making it necessary to prioritize head growth for sensorimotor coordination, predation capacity, and feeding success (Osse et al. 1997). Greater head development is typical in larvae of breeders or gametes subjected to environmental stress (Schoen et al. 2025; Coimbra et al. 2025).

Control larvae exhibited high yolk consumption only at 72 hph, confirming CAS-induced precocious metabolism. Conversely, CAS larvae displayed reduced body and notochord development, contrasting Pollock et al. (2005), in which *Archocentrus nigrofasciatus* offspring from CAS-exposed mothers showed enhanced somatic growth. However, other studies may help to understand our results. In McCormick’s study (1998), for example, maternal stress elevated cortisol levels in plasma, oocytes, and embryos, producing smaller larvae. Complementarily, in Lara and Vasconcelos’ study (2021), direct larval stress through noise resulted in smaller body size with rapid yolk depletion. Both studies indicate that stress, whether direct or indirect, can affect somatic growth and even yolk consumption in fish larvae.

The significantly reduced notochord length in larvae from CAS-exposed broodstock may indicate potential late-onset complications in locomotor and behavioral functions during danger situations, as it serves critical structural and mechanical roles in fish (Stemple 2005). The notochord standardizes the neural tube, specifies slow-twitch muscle contraction fates (Barresi et al. 2000; Devoto et al. 1996), forms the dorsal aorta (Cleaver et al. 2000; Fekany et al. 1999; Lawson et al. 2002), and establishes the cardiac field (Goldstein and Fishman 1998).

## Conclusion

The dual nature of stress and associated hormones on behavioral and reproductive aspects, whether stimulatory or inhibitory, limits precise assertions. In our previous study, the early onset of spawning in *A. bimaculatus* was abruptly interrupted; CAS-exposed broodstock spawned only once, whereas the control group spawned multiple times during the same event (Rodrigues et al. 2023). Furthermore, embryo and larval production was completely inhibited in the exposed group, resulting in zero survival, compared to over 10,000 viable larvae collected from the control group; here, spawning anticipation occurred, but produced morphometrically altered larvae. Thus, further investigations should be conducted to detect or rule out an intergenerational pattern triggered by CAS in this species. Acute exposure to CAS for evaluating reproductive performance in species requiring hormonal induction (Mechaly et al. 2023) may be suboptimal, potentially masked by the effects of the induction itself. Falahatkar and Poursaeid (2014) showed that induction with carp pituitary extract, by itself, elevates cortisol. Ord and Watt (2022) highlight the variability of the CAS extraction protocol and potentially ecologically irrelevant doses. All this underscores the urgent need for evaluations of this substance, especially in the reproductive context, as well as endocrine assessments to confirm its effect and a possible intergenerational pattern.

## Data Availability

The data that support the findings of this study are available from the corresponding author, [DHSS], upon reasonable request.
